# Supplementary material for: Antimicrobial Resistance in Commensal Bacteria from Large-Scale Chicken Flocks in the Dél-Alföld Region of Hungary
Source: Vet Sci. 2025 Jul 24;12(8):691. doi: 10.3390/vetsci12080691 (PMC12389816; doi:10.3390/vetsci12080691)
Supplement: Supplementary file 1 [file vetsci-12-00691-s001.zip › vetsci-3705989 - Supplementary materials.pdf]

Supplementary Table S1 Species identification of Staphylococcus isolates (n=41) isolated from poultry using MALDI-TOF

Number	MALDI-TOF	Log (score)
1	*Staphylococcus aureus ssp. aureus*	2.37
2	*Staphylococcus aureus ssp. aureus*	2.33
3	*Staphylococcus aureus ssp. aureus*	2.48
4	*Staphylococcus delphini*	2.45
5	*Staphylococcus aureus ssp. aureus*	2.32
6	*Staphylococcus gallinarum*	2.48
7	*Staphylococcus aureus ssp. aureus*	2.35
8	*Staphylococcus aureus ssp. aureus*	2.43
9	*Staphylococcus aureus ssp. aureus*	2.41
10	*Staphylococcus gallinarum*	2.20
11	*Staphylococcus aureus ssp. aureus*	2.32
12	*Staphylococcus delphini*	2.39
13	*Staphylococcus aureus ssp. aureus*	2.30
14	*Staphylococcus aureus ssp. aureus*	2.50
15	*Staphylococcus aureus ssp. aureus*	2.33
16	*Staphylococcus aureus ssp. aureus*	2.38
17	*Staphylococcus aureus ssp. aureus*	2.42
18	*Staphylococcus delphini*	2.49
19	*Staphylococcus gallinarum*	2.33
20	*Staphylococcus aureus ssp. aureus*	2.30
21	*Staphylococcus aureus ssp. aureus*	2.43
22	*Staphylococcus aureus ssp. aureus*	2.36
23	*Staphylococcus aureus ssp. aureus*	2.44
24	*Staphylococcus aureus ssp. aureus*	2.55
25	*Staphylococcus aureus ssp. aureus*	2.41
26	*Staphylococcus aureus ssp. aureus*	2.30
27	*Staphylococcus aureus ssp. aureus*	2.36
28	*Staphylococcus aureus ssp. aureus*	2.38
29	*Staphylococcus delphini*	2.42
30	*Staphylococcus aureus ssp. aureus*	2.30
31	*Staphylococcus aureus ssp. aureus*	2.34
32	*Staphylococcus delphini*	2.42
33	*Staphylococcus aureus ssp. aureus*	2.48
34	*Staphylococcus gallinarum*	2.44
35	*Staphylococcus aureus ssp. aureus*	2.45
36	*Staphylococcus aureus ssp. aureus*	2.40
37	*Staphylococcus aureus ssp. aureus*	2.42
38	*Staphylococcus aureus ssp. aureus*	2.39
39	*Staphylococcus aureus ssp. aureus*	2.30
40	*Staphylococcus aureus ssp. aureus*	2.32
41	*Staphylococcus aureus ssp. aureus*	2.45

2.00 – 3.00 score: High Confidence Identification

Supplementary Table S2 Species identification of Enterococcus isolates (n=59) isolated from poultry using MALDI-TOF

Number	MALDI-TOF	Log (score)
1	*Enterococcus faecium*	2.31
2	*Enterococcus faecium*	2.29
3	*Enterococcus faecalis*	2.46
4	*Enterococcus faecium*	2.42
5	*Enterococcus faecium*	2.22
6	*Enterococcus faecalis*	2.33
7	*Enterococcus faecalis*	2.31
8	*Enterococcus faecium*	2.49
9	*Enterococcus faecium*	2.44
10	*Enterococcus faecium*	2.25
11	*Enterococcus faecium*	2.36
12	*Enterococcus faecalis*	2.37
13	*Enterococcus durans*	2.21
14	*Enterococcus faecium*	2.11
15	*Enterococcus durans*	2.09
16	*Enterococcus faecium*	2.07
17	*Enterococcus faecium*	2.15
18	*Enterococcus faecalis*	2.63
19	*Enterococcus durans*	2.78
20	*Enterococcus durans*	2.55
21	*Enterococcus faecium*	2.16
22	*Enterococcus faecium*	2.12
23	*Enterococcus faecalis*	2.46
24	*Enterococcus durans*	2.57
25	*Enterococcus hirae*	2.42
26	*Enterococcus durans*	2.33
27	*Enterococcus mundtii*	2.38
28	*Enterococcus faecalis*	2.39
29	*Enterococcus faecalis*	2.45
30	*Enterococcus faecalis*	2.21
31	*Enterococcus faecalis*	2.13
32	*Enterococcus faecalis*	2.71
33	*Enterococcus faecalis*	2.81
34	*Enterococcus faecalis*	2.33
35	*Enterococcus gallinarum*	2.55
36	*Enterococcus hirae*	2.54
37	*Enterococcus gallinarum*	2.14
38	*Enterococcus faecalis*	2.21
39	*Enterococcus faecalis*	2.16
40	*Enterococcus faecium*	2.41
41	*Enterococcus faecium*	2.46
42	*Enterococcus faecium*	2.77
43	*Enterococcus faecalis*	2.14
44	*Enterococcus faecalis*	2.52

45	*Enterococcus faecalis*	2.31
46	*Enterococcus faecalis*	2.47
47	*Enterococcus gallinarum*	2.15
48	*Enterococcus faecalis*	2.17
49	*Enterococcus faecalis*	2.18
50	*Enterococcus faecalis*	2.54
51	*Enterococcus faecalis*	2.34
52	*Enterococcus faecalis*	2.54
53	*Enterococcus faecalis*	2.18
54	*Enterococcus faecium*	2.22
55	*Enterococcus faecium*	2.23
56	*Enterococcus faecium*	2.41
57	*Enterococcus faecium*	2.30
58	*Enterococcus faecium*	2.16
59	*Enterococcus faecalis*	2.19

2.00 – 3.00 score: High Confidence Identification

Supplementary Table S3 Species identification of Escherichia coli isolates (n=45) isolated from poultry using MALDI-TOF

Number	MALDI-TOF	Log (score)
1	*Escherichia coli*	2.42
2	*Escherichia coli*	2.26
3	*Escherichia coli*	2.32
4	*Escherichia coli*	2.29
5	*Escherichia coli*	2.50
6	*Escherichia coli*	2.47
7	*Escherichia coli*	2.22
8	*Escherichia coli*	2.21
9	*Escherichia coli*	2.30
10	*Escherichia coli*	2.29
11	*Escherichia coli*	2.30
12	*Escherichia coli*	2.29
13	*Escherichia coli*	2.45
14	*Escherichia coli*	2.43
15	*Escherichia coli*	2.30
16	*Escherichia coli*	2.28
17	*Escherichia coli*	2.30
18	*Escherichia coli*	2.28
19	*Escherichia coli*	2.37
20	*Escherichia coli*	2.32
21	*Escherichia coli*	2.49
22	*Escherichia coli*	2.47
23	*Escherichia coli*	2.42
24	*Escherichia coli*	2.37
25	*Escherichia coli*	2.30
26	*Escherichia coli*	2.27

27	*Escherichia coli*	2.58
28	*Escherichia coli*	2.45
29	*Escherichia coli*	2.36
30	*Escherichia coli*	2.33
31	*Escherichia coli*	2.13
32	*Escherichia coli*	2.05
33	*Escherichia coli*	2.36
34	*Escherichia coli*	2.35
35	*Escherichia coli*	2.14
36	*Escherichia coli*	2.12
37	*Escherichia coli*	2.22
38	*Escherichia coli*	2.19
39	*Escherichia coli*	2.32
40	*Escherichia coli*	2.28
41	*Escherichia coli*	2.27
42	*Escherichia coli*	2.26
43	*Escherichia coli*	2.24
44	*Escherichia coli*	2.24
45	*Escherichia coli*	2.07

2.00 – 3.00 score: High Confidence Identification

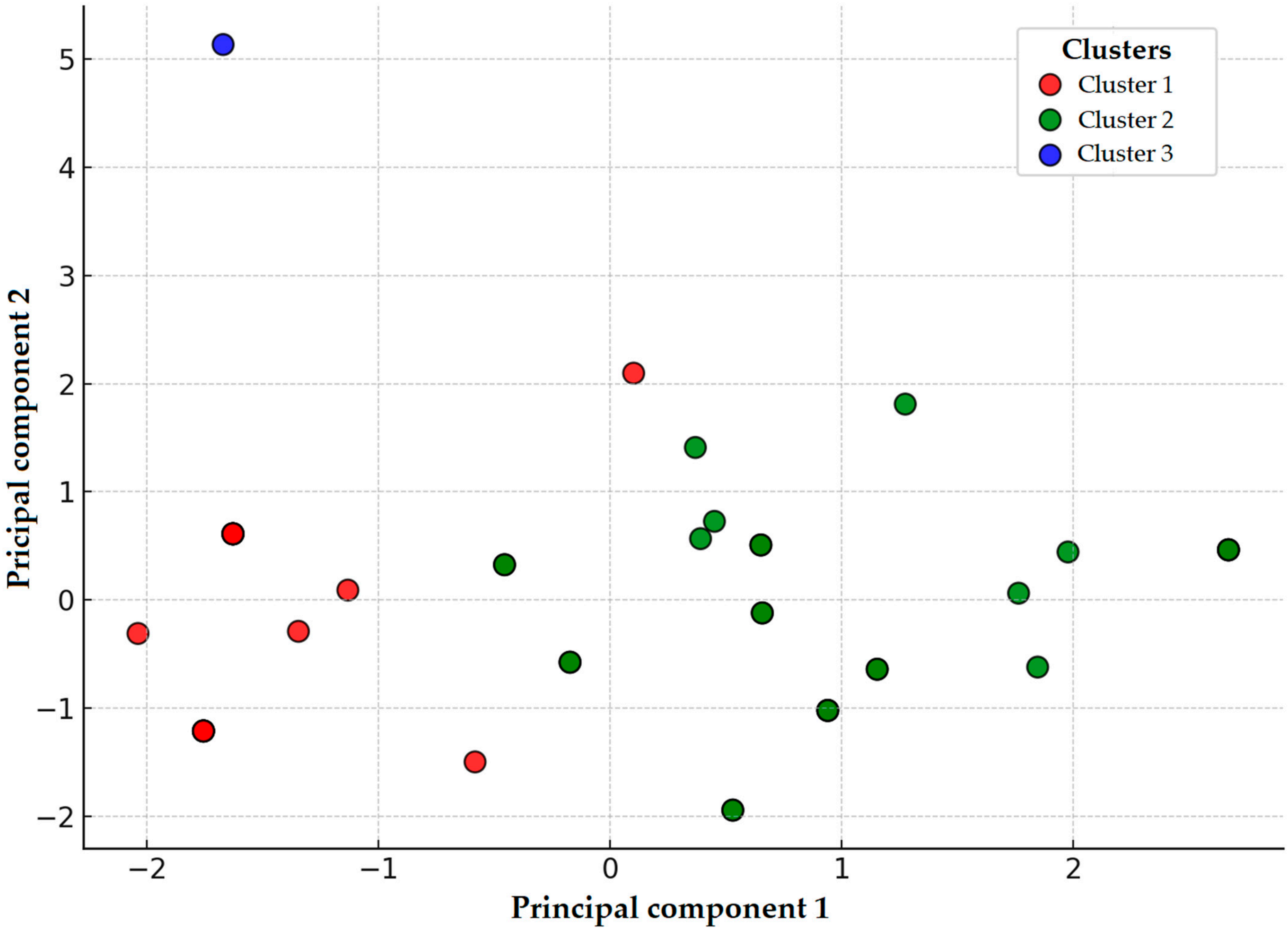

Supplementary Figure S1 Principal component analysis (PCA) and clustering of *Staphylococcus* spp. isolates (*n*=41) isolated from chickens in the Dél-Alföld region. Clusters 1, 2, and 3 are indicated by the colors red, green, and blue, respectively.

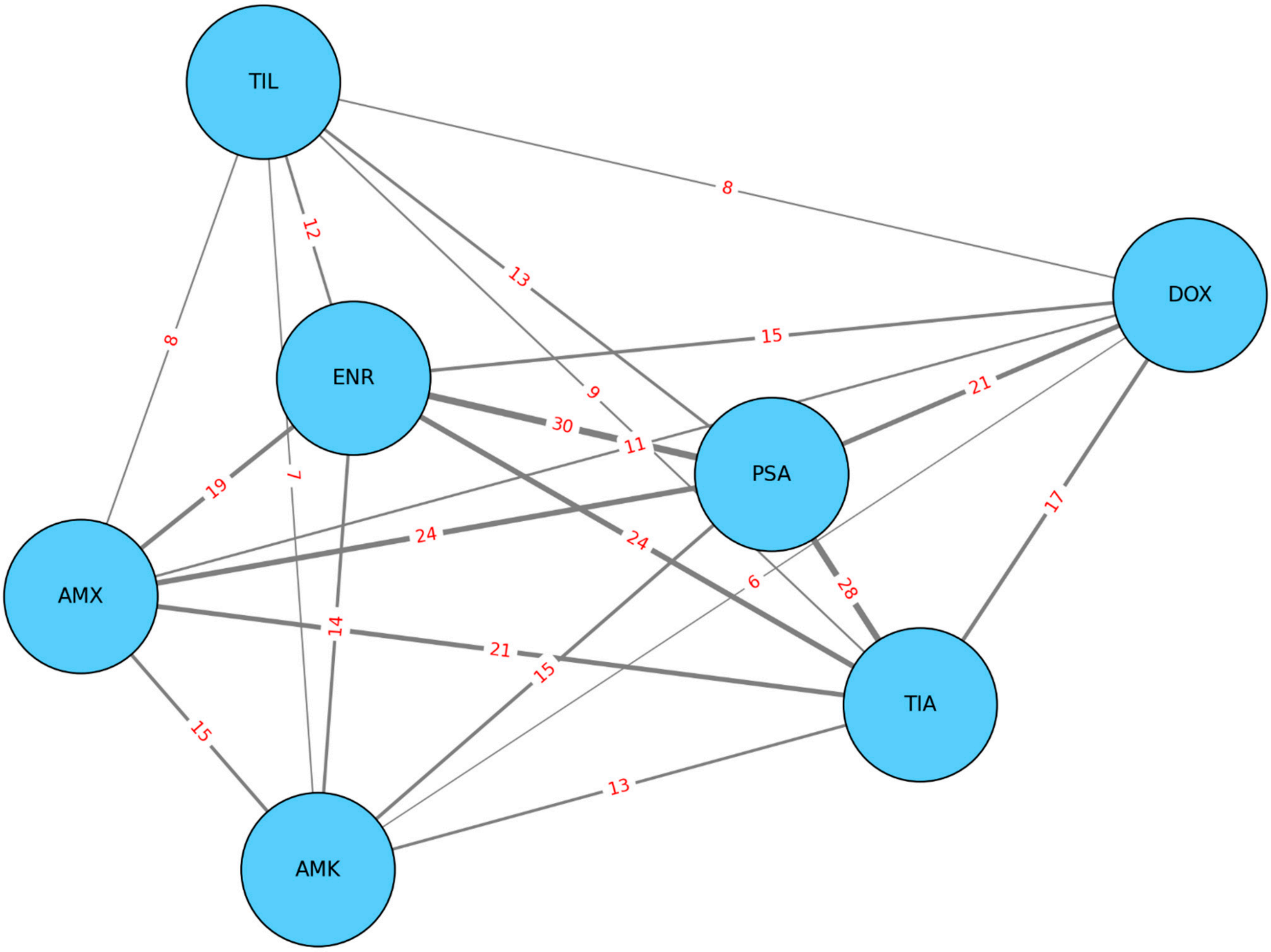

Supplementary Figure S2 Resistance-based network graph of *Staphylococcus* spp. isolates (*n*=41) isolated from chickens in the Dél-Alföld region. The strongest associations were observed between enrofloxacin and potentiated sulfonamide, as well as between enrofloxacin and tiamulin. AMX – amoxicillin; AMK – amoxicillin-clavulanic acid; DOX – doxycycline; TIL – tylosin; TIA – tiamulin; ENR – enrofloxacin; PSA – potentiated sulfonamide (trimethoprim-sulfamethoxazole, 1:19)

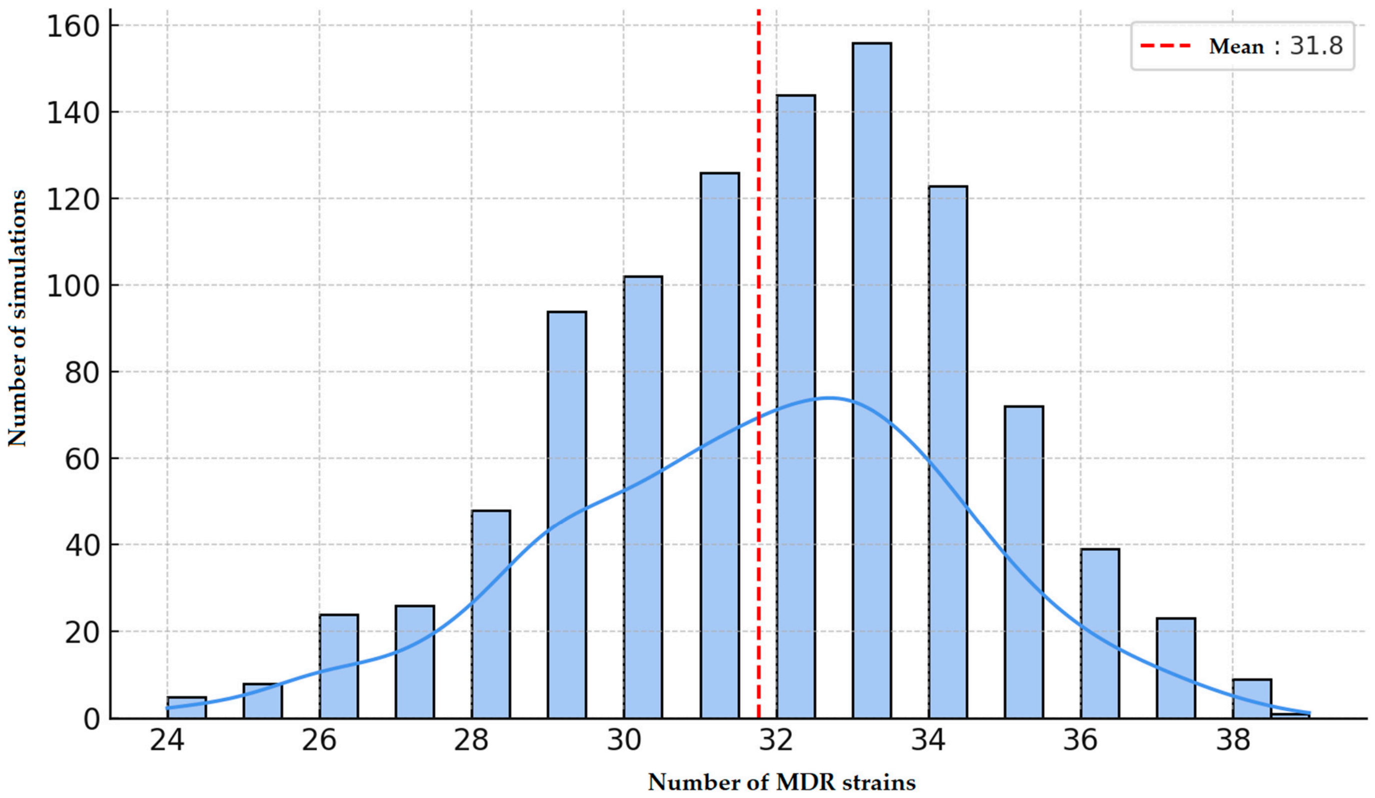

Supplementary Figure S3 Stochastic estimation of the occurrence of multidrug-resistant (MDR) *Staphylococcus* spp. isolates isolated from chickens in the Dél-Alföld region using Monte Carlo simulation.

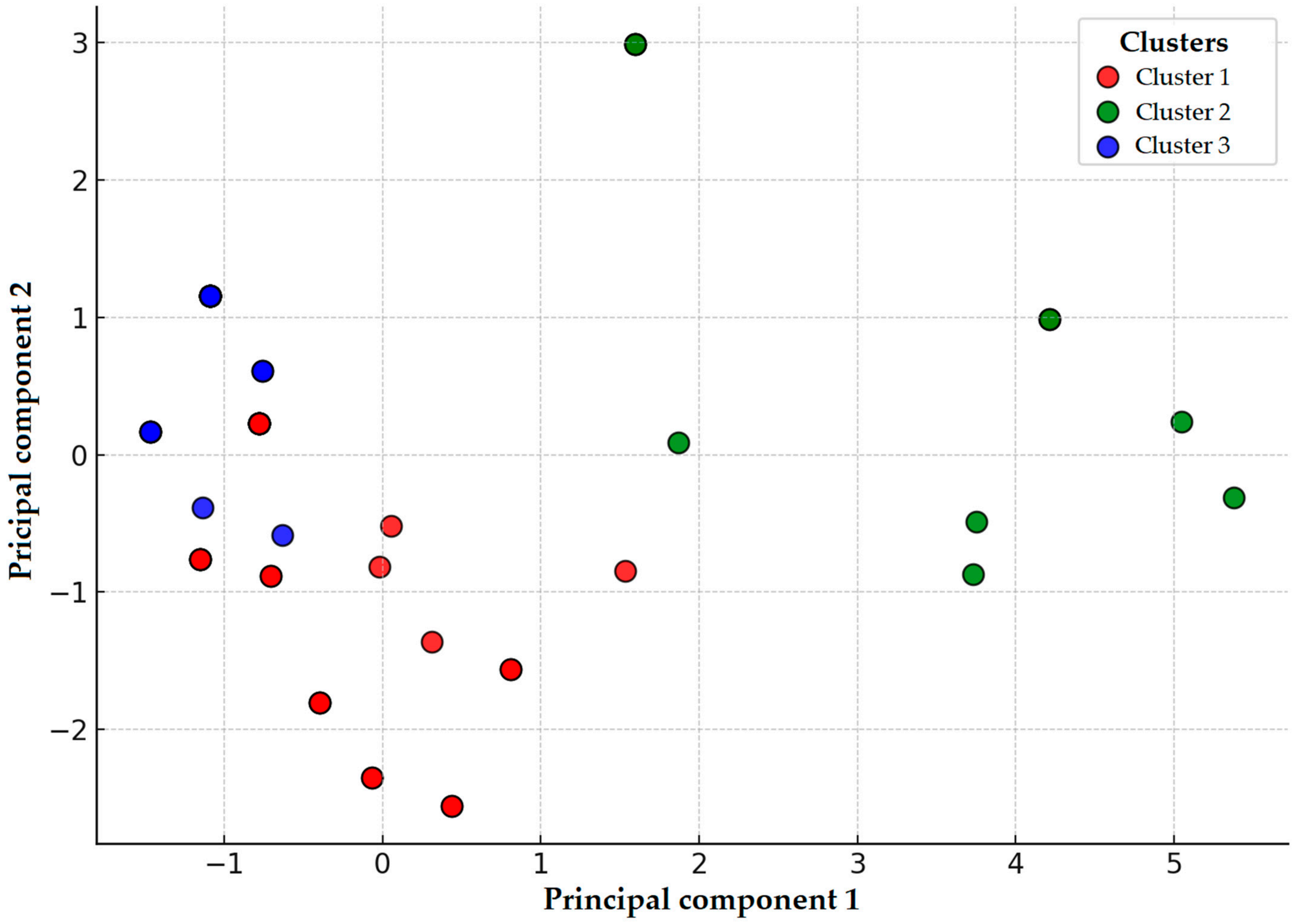

Supplementary Figure S4 Principal component analysis (PCA) and clustering of *Enterococcus* spp. isolates (n=59) isolated from chickens in the Dél-Alföld region. Clusters 1, 2, and 3 are indicated by the colors red, green, and blue, respectively.

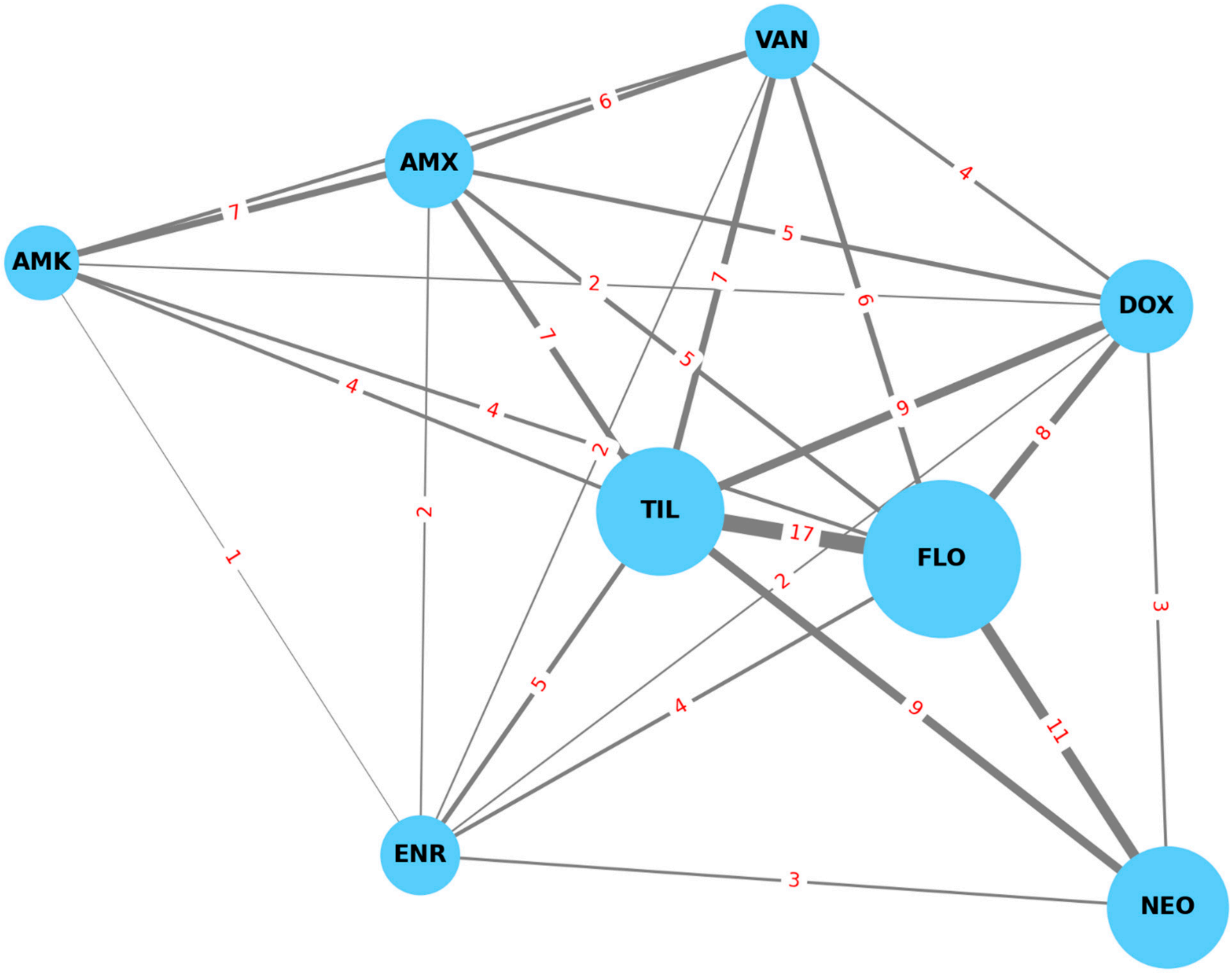

Supplementary Figure S5 Resistance-based network graph of *Enterococcus* spp. isolates (*n*=59) isolated from chickens in the Dél-Alföld region. The strongest associations were observed among florfenicol, neomycin, and tylosin. AMX – amoxicillin; AMK – amoxicillin-clavulanic acid; NEO – neomycin; DOX – doxycycline; FLO – florfenicol; TIL – tylosin; ENR – enrofloxacin; VAN – vancomycin.

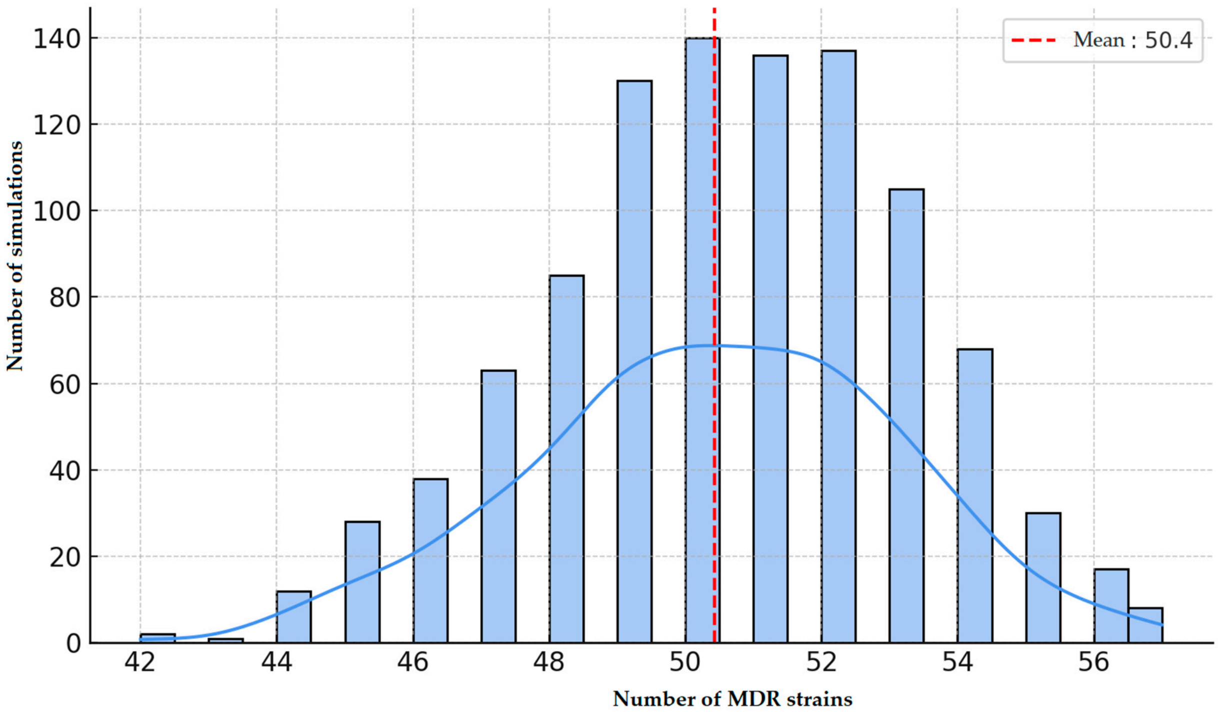

Supplementary Figure S6 Monte Carlo-based stochastic estimation of multidrug-resistant (MDR) *Enterococcus* spp. isolates isolated from chickens in the Dél-Alföld region.

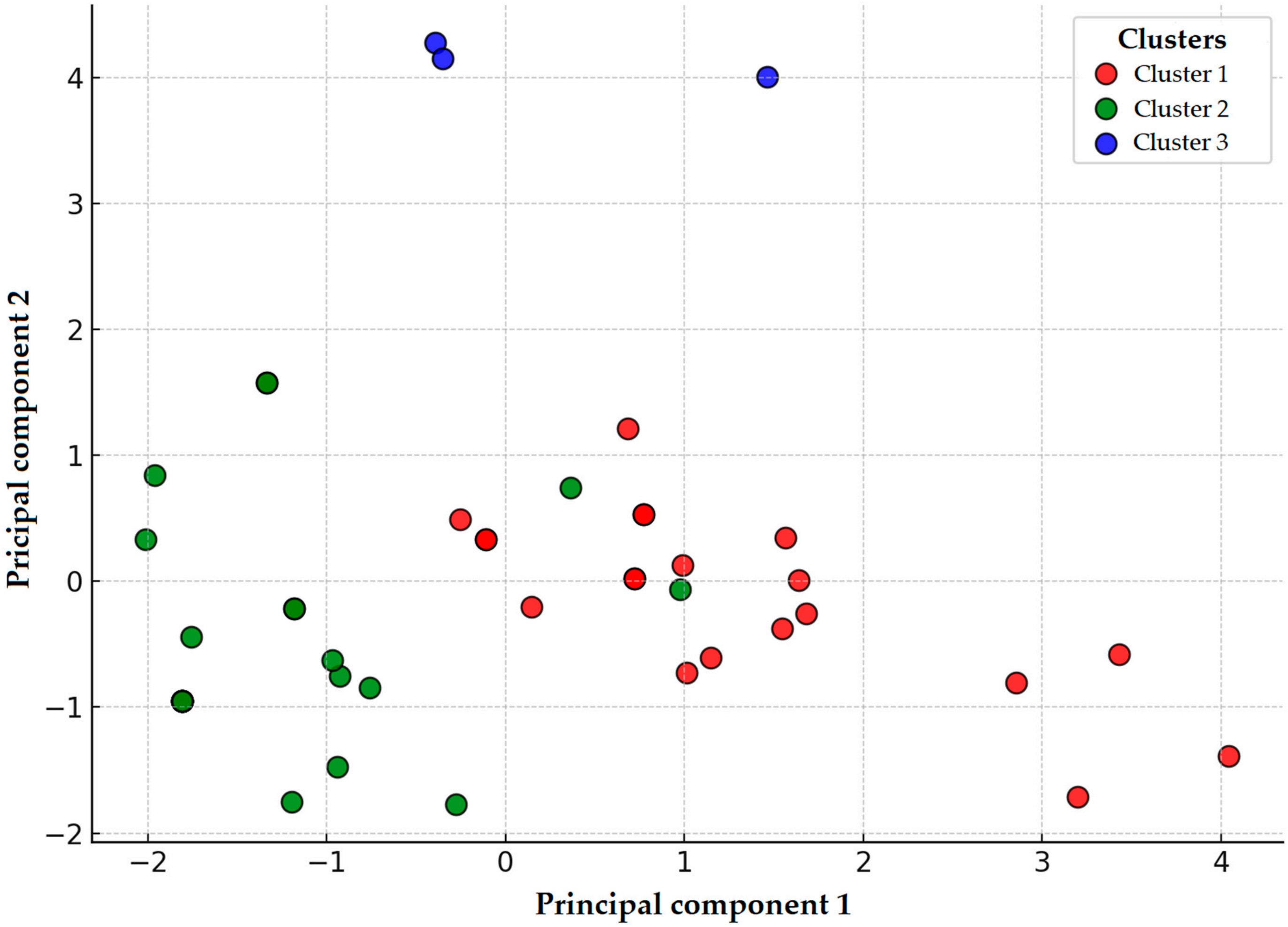

Supplementary Figure S7 PCA and clustering of *Escherichia coli* isolates (n=45) isolated from chickens in the Dél-Alföld region. Clusters 1, 2, and 3 are indicated by the colors red, green, and blue, respectively.

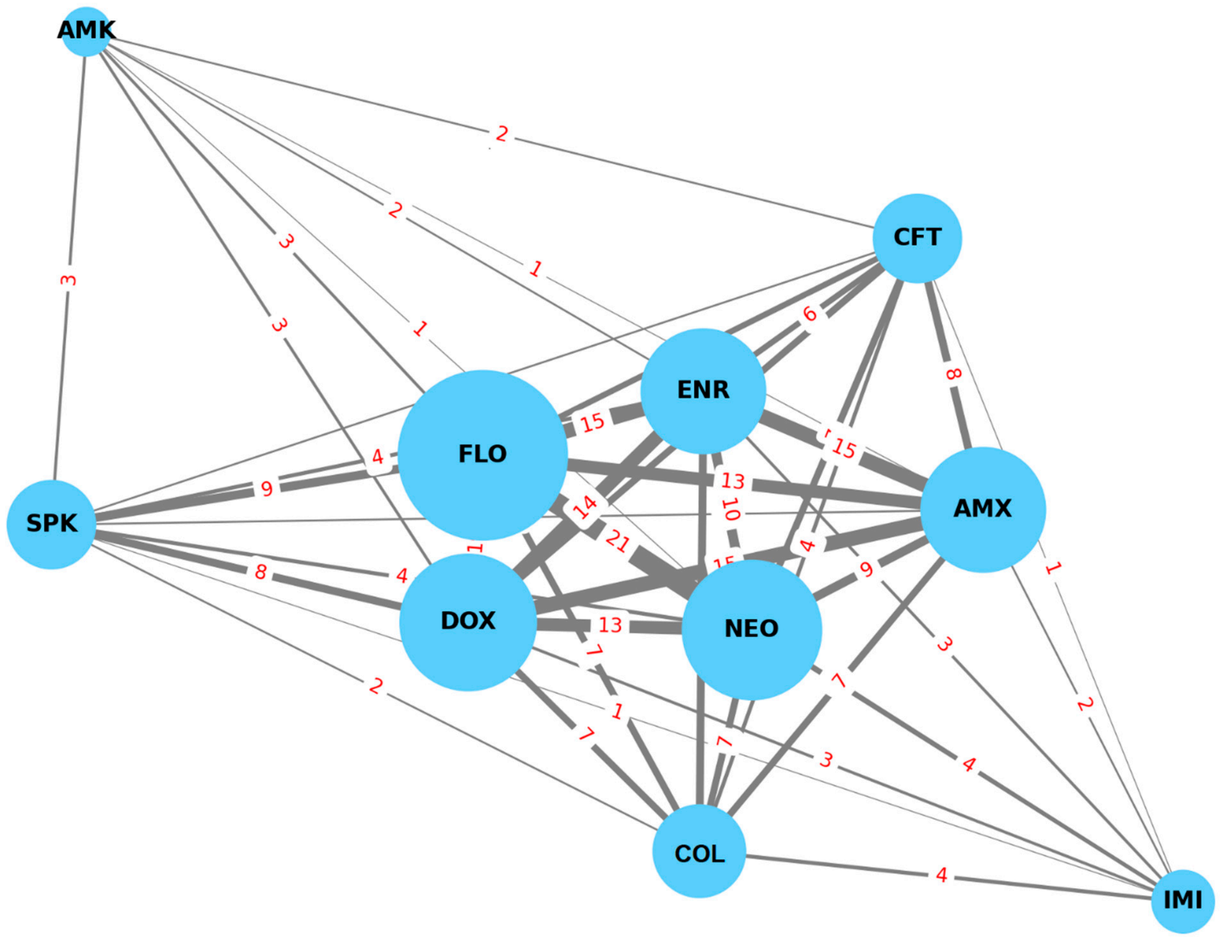

Supplementary Figure S8 Resistance-based network graph of *Escherichia coli* isolates (*n*=45) isolated from chickens in the Dél-Alföld region. The strongest associations were found among florfenicol, neomycin, and enrofloxacin. AMX – amoxicillin; AMK – amoxicillin-clavulanic acid; CFT – ceftriaxone; NEO – neomycin; SPK – spectinomycin; DOX – doxycycline; FLO – florfenicol; ENR – enrofloxacin; IMI – imipenem; COL – colistin.

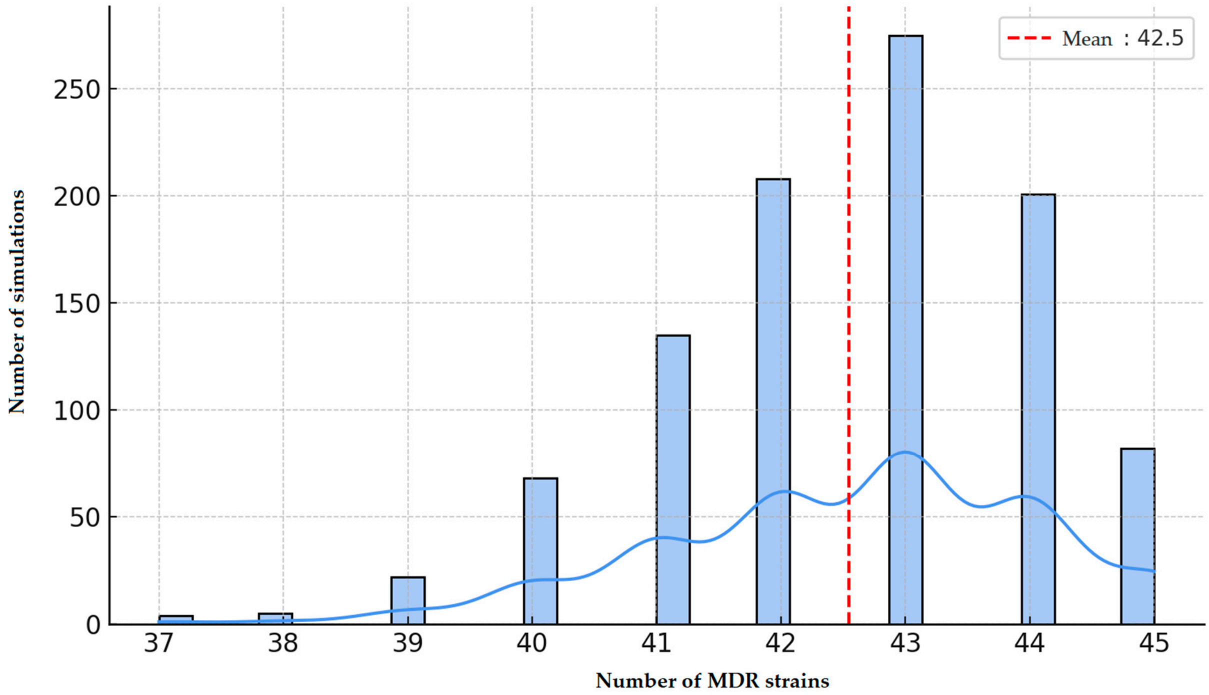

Supplementary Figure S9 Monte Carlo simulation-based stochastic estimation of the occurrence of multidrug-resistant (MDR) *Escherichia coli* isolates in the Dél-Alföld region.
