# Supplementary figures and images for: Antimicrobial Resistance in Commensal Bacteria from Large-Scale Chicken Flocks in the Dél-Alföld Region of Hungary
Source: Vet Sci. 2025 Jul 24;12(8):691. doi: 10.3390/vetsci12080691 (PMC12389816; doi:10.3390/vetsci12080691)

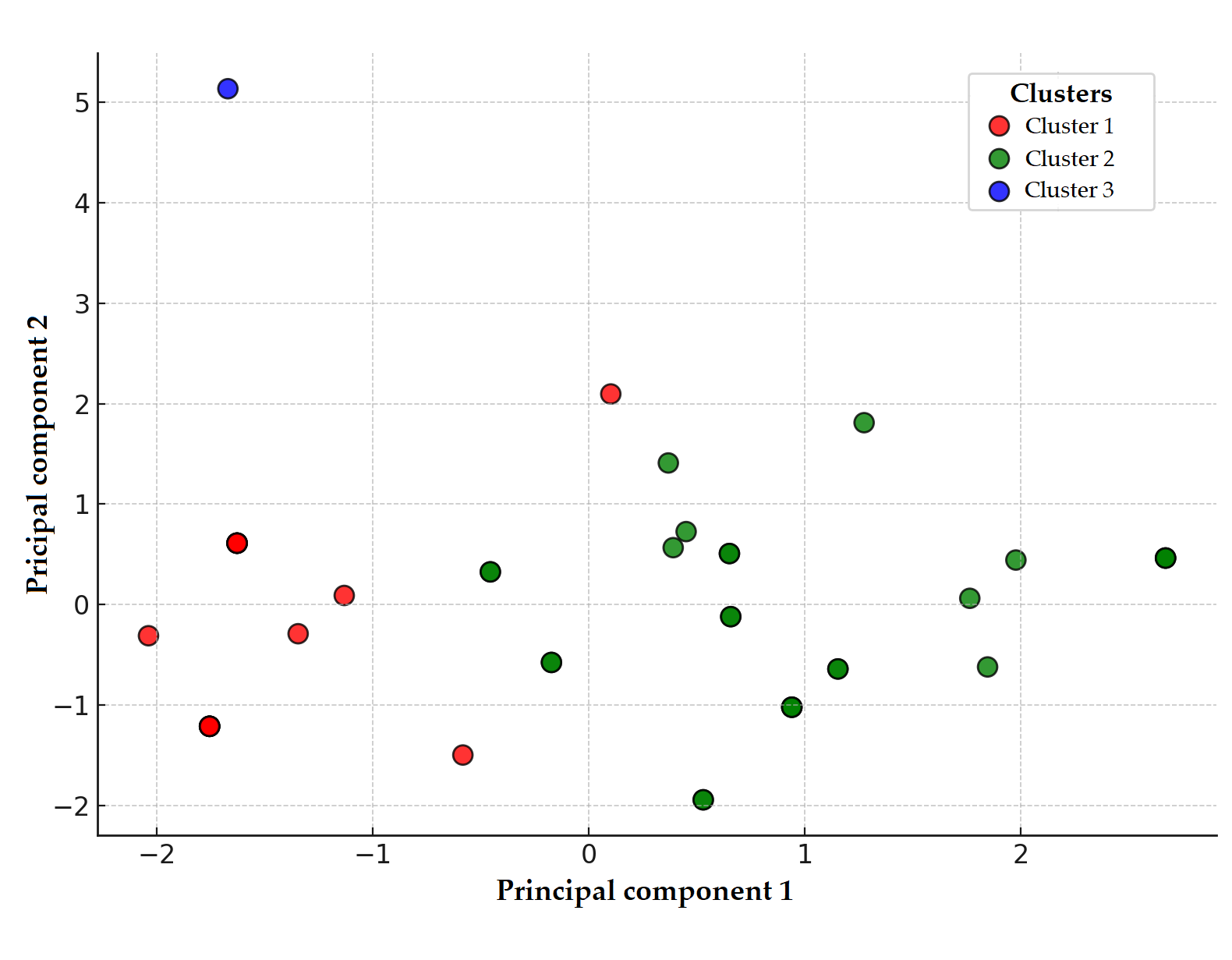

Supplement: Supplementary file 1 [file vetsci-12-00691-s001.zip › Supplementary Figure S1.png]

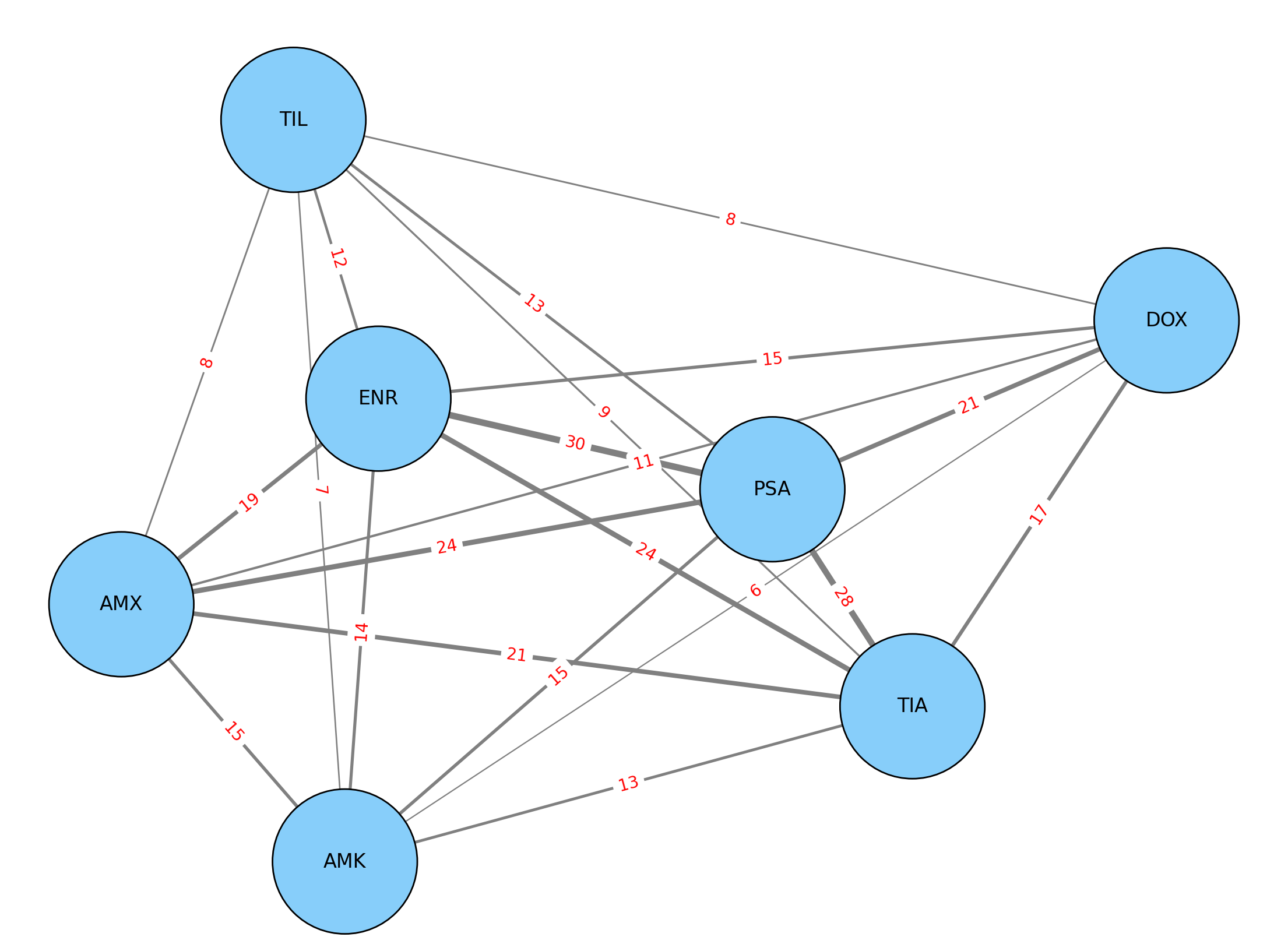

Supplement: Supplementary file 1 [file vetsci-12-00691-s001.zip › Supplementary Figure S2.png]

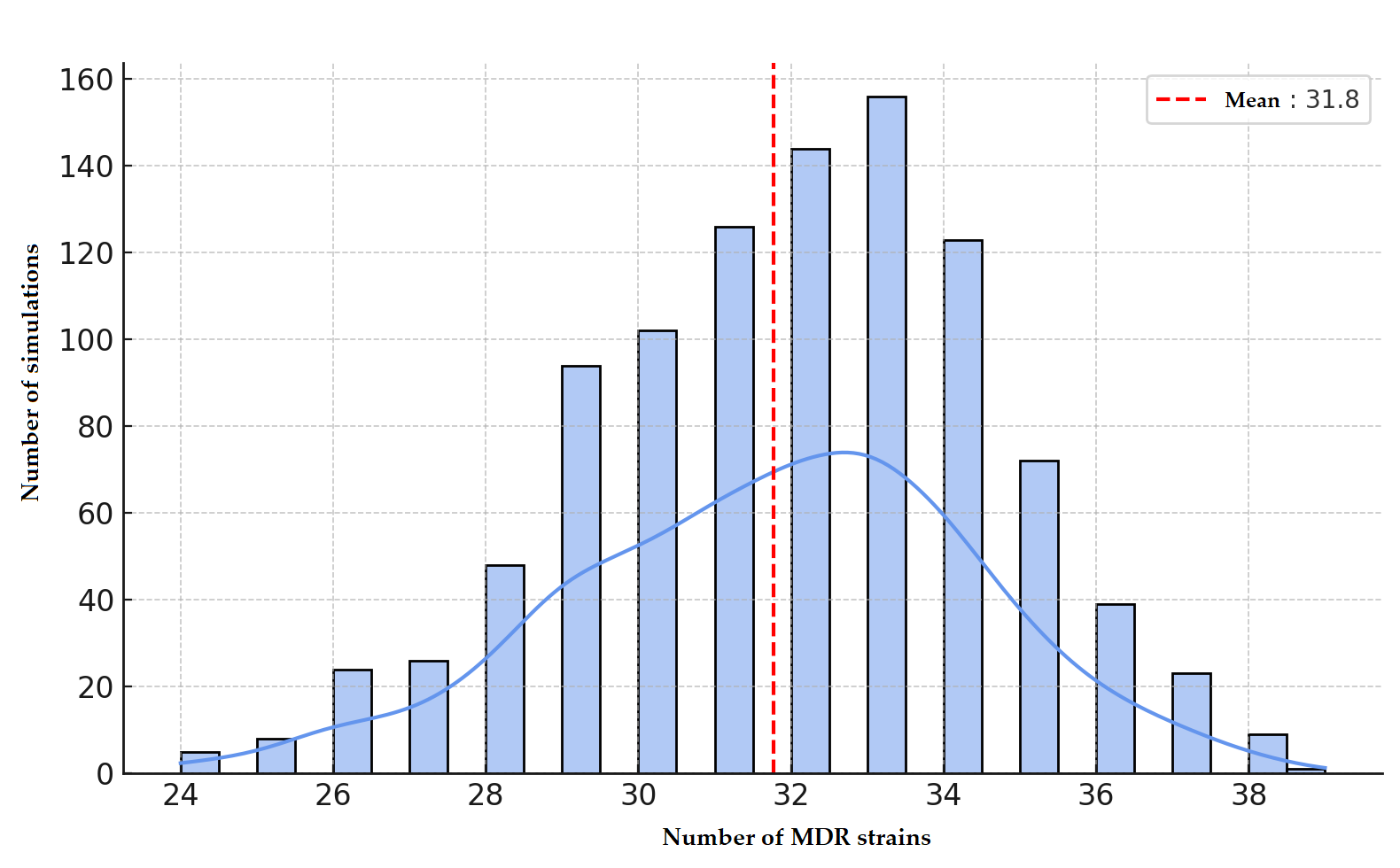

Supplement: Supplementary file 1 [file vetsci-12-00691-s001.zip › Supplementary Figure S3.png]

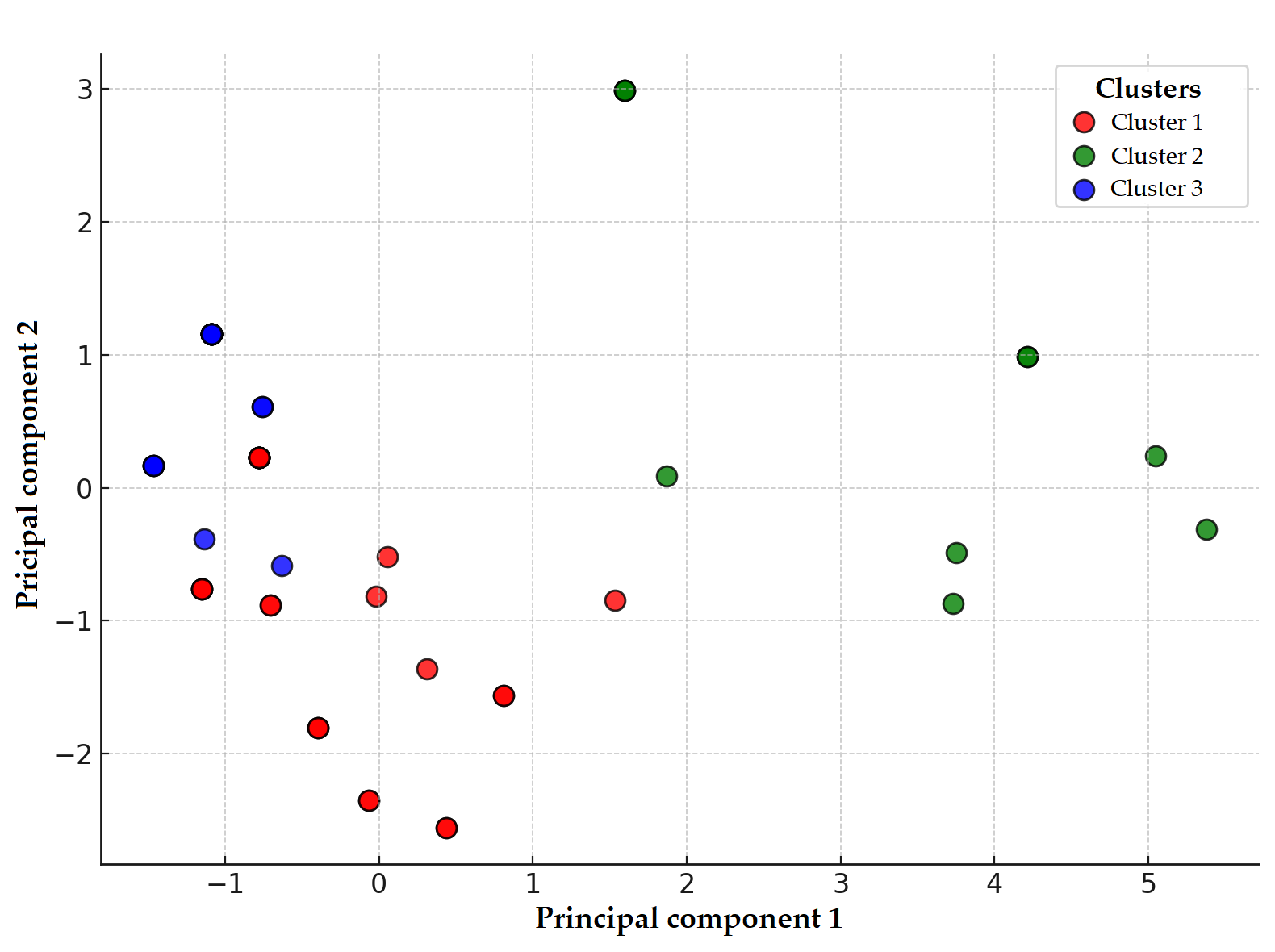

Supplement: Supplementary file 1 [file vetsci-12-00691-s001.zip › Supplementary Figure S4.png]

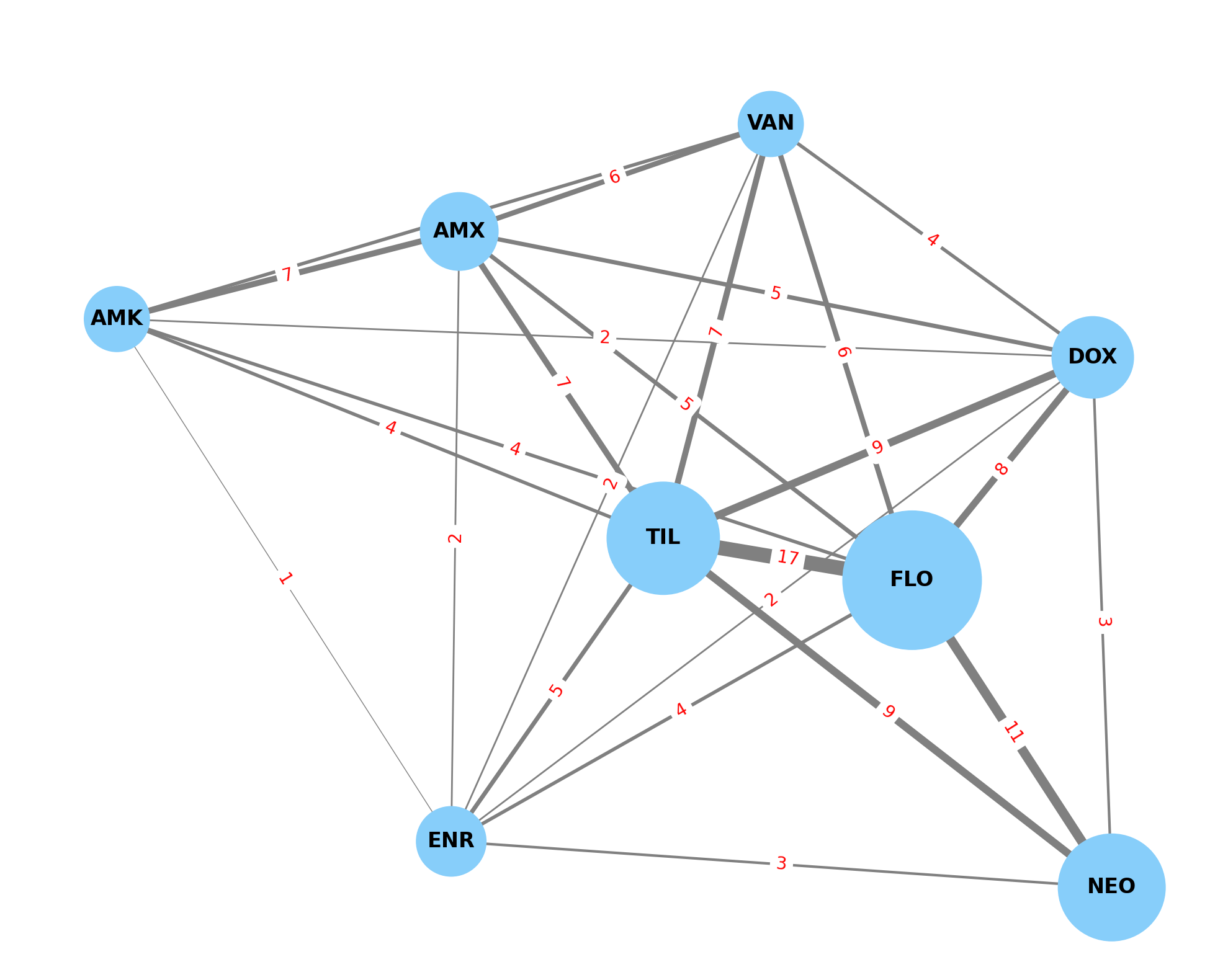

Supplement: Supplementary file 1 [file vetsci-12-00691-s001.zip › Supplementary Figure S5.png]

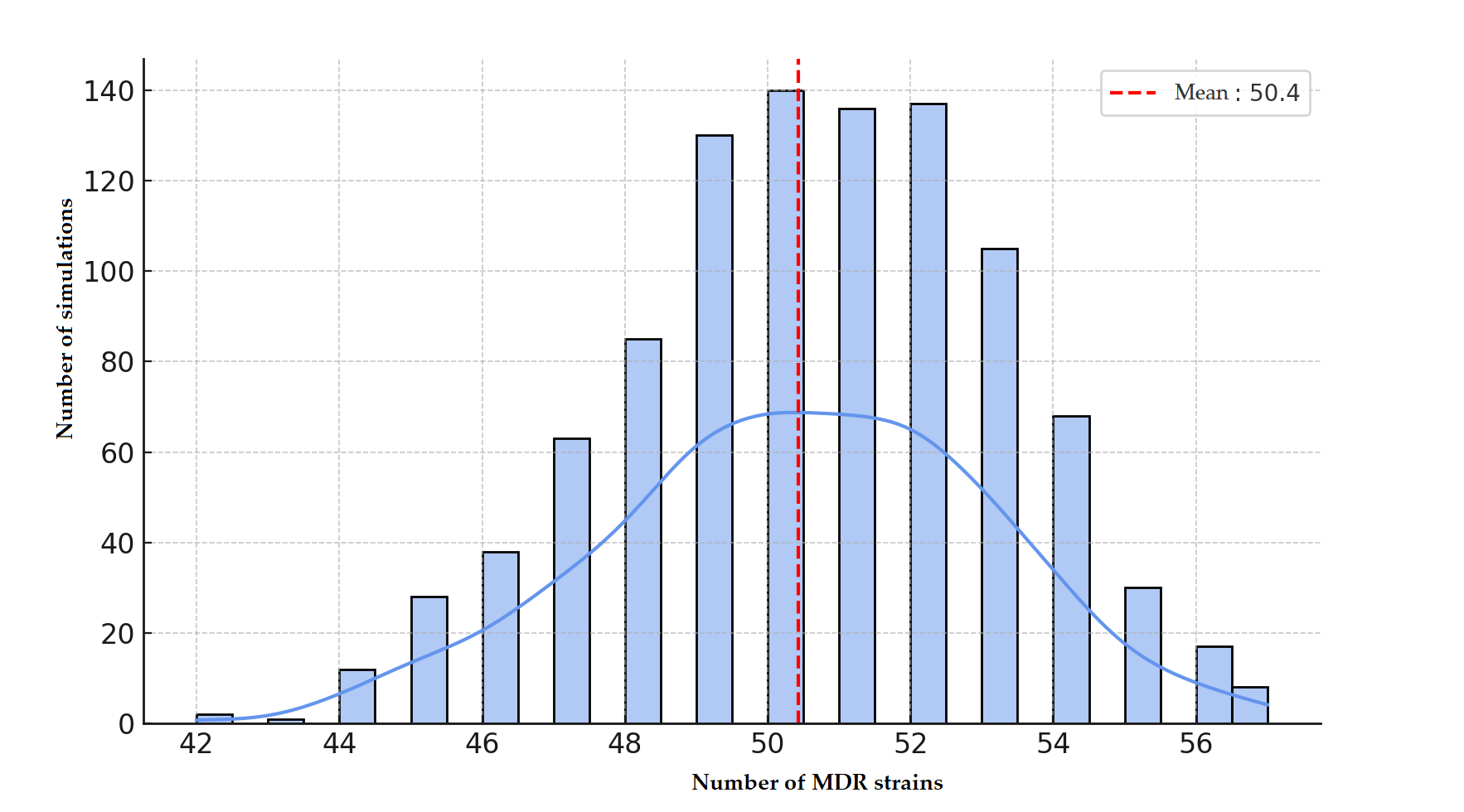

Supplement: Supplementary file 1 [file vetsci-12-00691-s001.zip › Supplementary Figure S6.png]

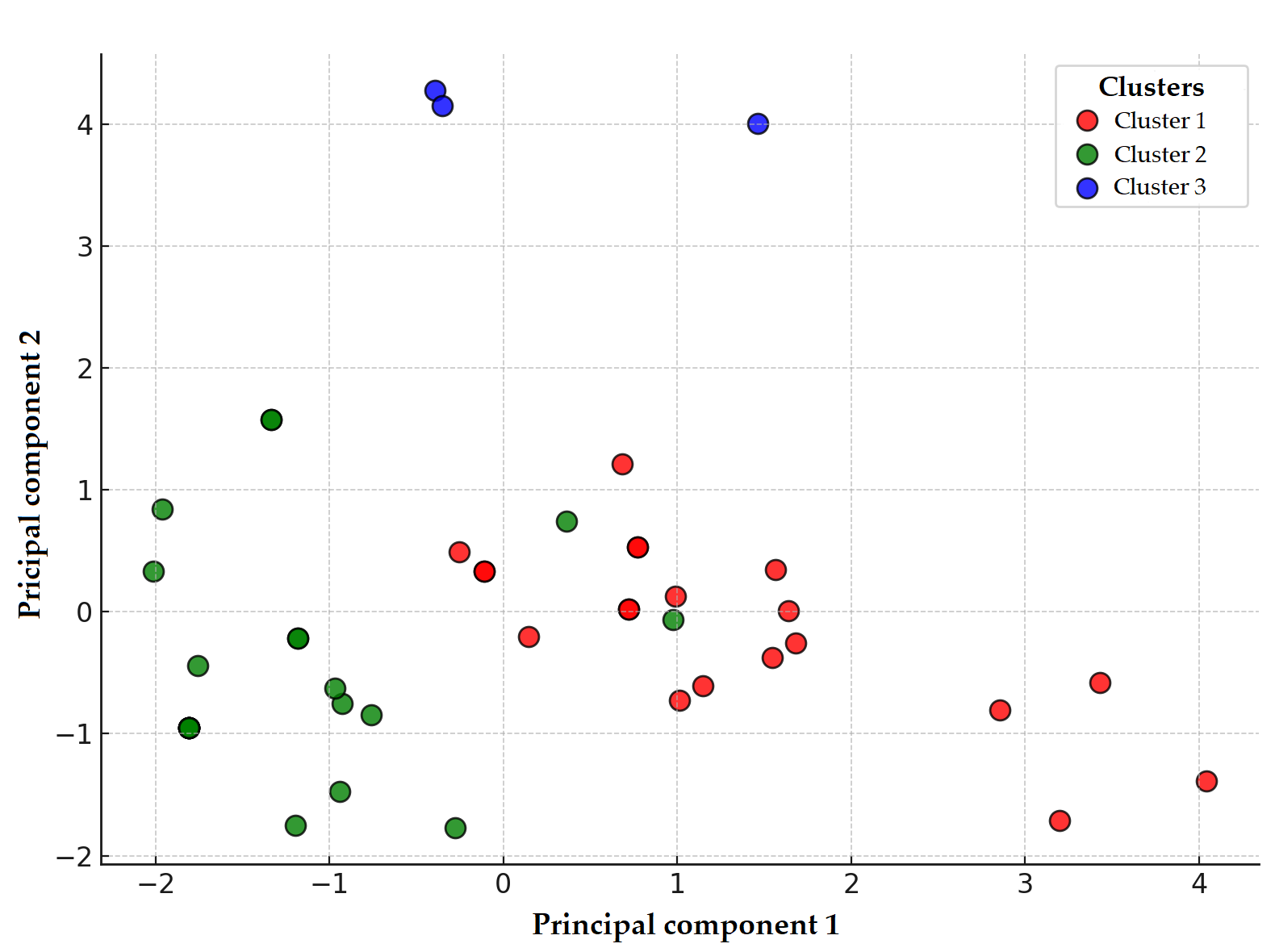

Supplement: Supplementary file 1 [file vetsci-12-00691-s001.zip › Supplementary Figure S7.png]

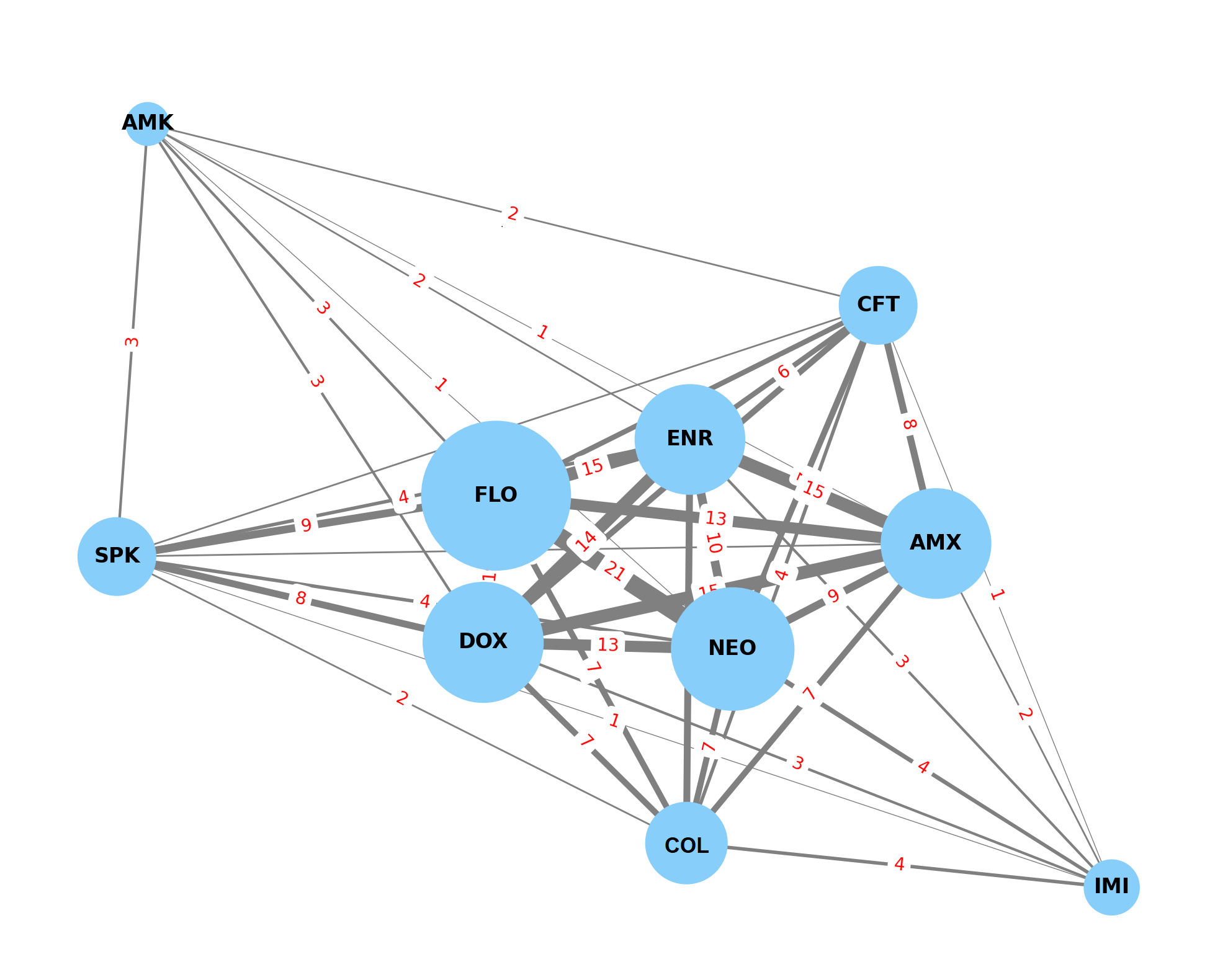

Supplement: Supplementary file 1 [file vetsci-12-00691-s001.zip › Supplementary Figure S8.png]

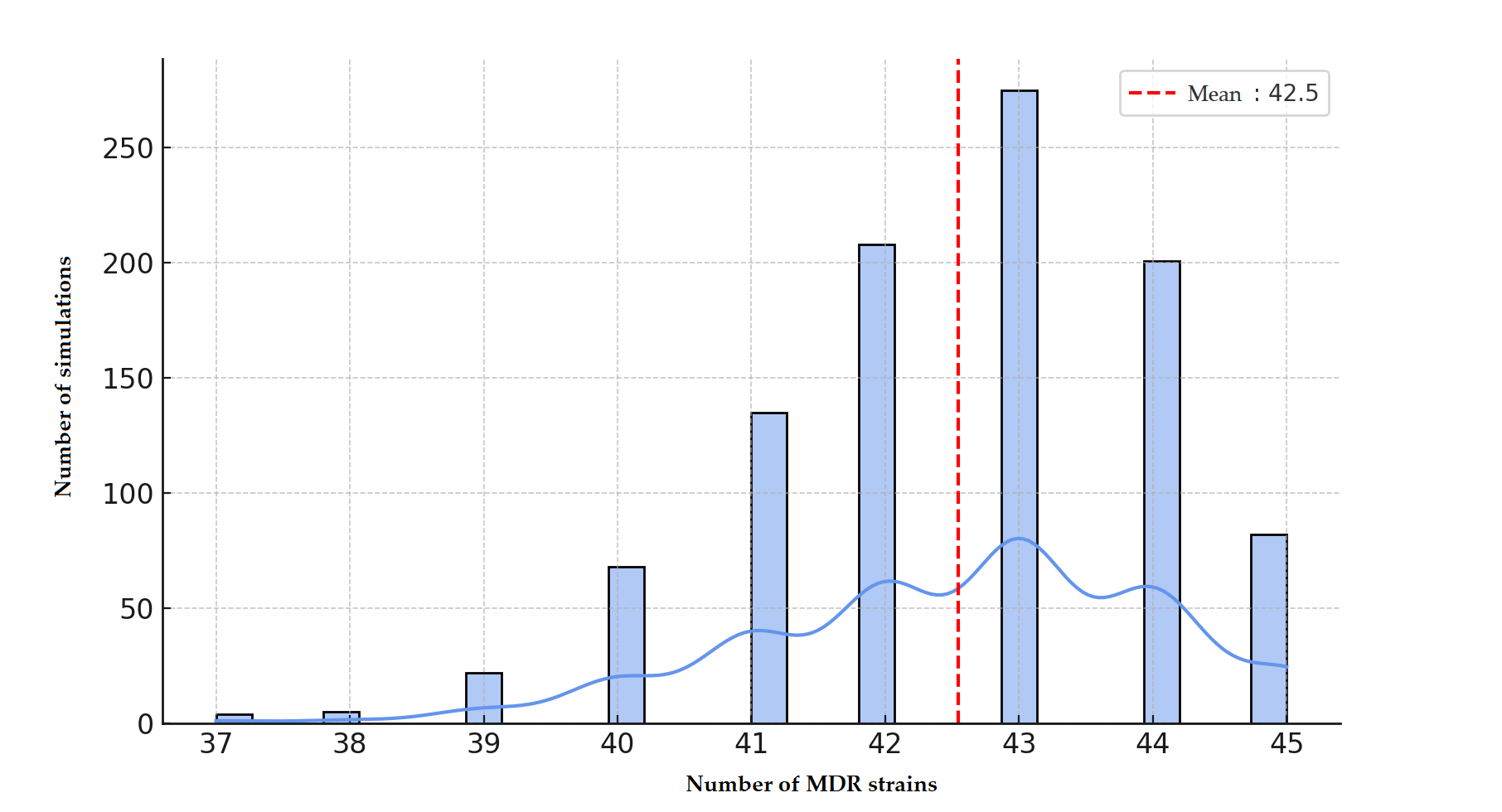

Supplement: Supplementary file 1 [file vetsci-12-00691-s001.zip › Supplementary Figure S9.png]
